# Spanish validation of the Modified Weight Bias Internalization Scale (WBIS-M) for adolescents

**DOI:** 10.1007/s40519-022-01453-z

**Published:** 2022-07-28

**Authors:** Ana Andrés, Albert Fornieles-Deu, Ana Rosa Sepúlveda, Lucía Beltrán-Garrayo, Albert Montcada-Ribera, Anna Bach-Faig, David Sánchez-Carracedo

**Affiliations:** 1 Faculty of Psychology, Education and Sport Sciences, Blanquerna, Ramon Llull University, Barcelona, Spain; 2 Eating and Weight-Related Problems Unit, Universitat Autònoma de Barcelona, 08193 Barcelona, Spain; 3 Department of Psychobiology and Methodology of Health Sciences, Serra Hunter Fellow, Universitat Autònoma de Barcelona, 08193 Barcelona, Spain; 4 Department of Biological and Health Psychology, School of Psychology, Autonomous University of Madrid, 28049 Madrid, Spain; 5 Head of Community and Health Service, City Council of Terrassa, Social Rights Section, Barcelona, Spain; 6 FoodLab Research Group (2017SGR 83, Faculty of Health Sciences, Universitat Oberta de Catalunya (Open University of Catalonia, UOC), 08018 Barcelona, Spain; 7 Food and Nutrition Area, Barcelona Official College of Pharmacists, 08009 Barcelona, Spain; 8 Department of Clinical and Health Psychology, Universitat Autònoma de Barcelona, 08193 Barcelona, Spain

**Keywords:** Confirmatory factor analysis, Exploratoy factor analysis, Weight Bias Internalization, Obesity stigma, Adolescents

## Abstract

**Purpose:**

Weight Bias Internalization (WBI) is pervasive and potentially damaging for health. Little is known about WBI in youth. As negative effects of WBI have been observed when controlling for BMI, measures that allow WBI to be assessed across different weight categories are needed. The Modified Weight Bias Internalization Scale (WBIS-M) is one of the most frequently used scales in this field. Our purpose was to obtain a Spanish validated version of the WBIS-M for adolescents across different weight statuses.

**Methods:**

The data were collected from 298 secondary students (mean age 14.31; 48.32% girls; 18.8% were overweight and 6.4% had obesity). Internal structure was examined by a cross-validation analysis, using both exploratory and confirmatory factor analyses in different subsamples.

**Results:**

Item 1 showed a psychometric anomalous functioning and was deleted. The one-factor structure of the 10-item version was confirmed with adequate fit ([EFA (KMO = 0.915, *χ*^2^_(55)_ = 1075.633, *p* < 0.0001)]; [CFA (*χ*^2^_(35)_ = 200.515; GFI = 0.995; PGFI = 0.992; NFI = 0.991; SRMR = 0.060)]). Internal consistency was high $(\alpha =0.93;$ *ω* = 0.93). Significant correlations with the same set of external variables assessed in the original version (anti-fat bias, self-esteem, mood, body dissatisfaction, drive for thinness, binge eating), all of them correlates of WBI in adolescents, were found. Girls and participants with obesity obtained higher scores.

**Conclusion:**

The results provide support for the validity and reliability of our WBIS-M version for use with adolescents across weight categories in Spain.

**Level of evidence:**

Level III, evidence obtained from well-designed cohort studies.

## Introduction

Obesity stigma, also known as weight stigma and weight bias, is pervasive in Western societies and has been defined as a globalizing health challenge [1]. Weight stigma refers to social devaluation and denigration of individuals because of their excess body weight, and can lead to negative attitudes, stereotypes, prejudice, and discrimination [2]. Weight bias, when explicit, refers to overt, consciously held negative attitudes, while implicit weight bias refers to automatic, negative attributions and stereotypes which exist outside of conscious awareness [2].

Weight stigma has been associated with psychological distress [3], poorer mental health [4], disordered eating and unhealthy eating behaviours [5], substance use [6], more physiological stress [7], reduced motivation to engage in physical activity [8], additional barriers to weight loss maintenance [9], reduced healthcare seeking behaviour [10], and a higher risk for suicidality and mortality [11, 12].

Because weight stigma negatively affects weight-related behaviours and health, it can, paradoxically, contribute to worsened problems associated with obesity and create additional barriers to healthy behaviour change [13]. Therefore, weight stigma is considered a psychosocial contributor to obesity [14]. A recent meta-analysis has supported bidirectional relationships between weight stigma and paediatric obesity [15].

*Weight bias internalization* (WBI) occurs when individuals engage in self-blame and self-directed weight stigma because of their weight [2]. WBI is also pervasive and potentially damaging for health beyond body weight and experiences of stigma [16, 17], leading to increased research attention in recent years, especially in adults. In contrast, not much is known about WBI in youth [18]. Children and adolescents can be particularly vulnerable to weight stigma, with long-lasting consequences that negatively affect their life course and emotional and physical well being [6]. Thus, tools for assessing experienced and internalized weight bias in children and adolescents are needed as a first step to study their negative impact, and as a measure outcome in future studies focused on how to address weight bias.

A systematic review [19] identified two questionnaires designed to assess WBI in people who are overweight or have obesity: the Weight Self-Stigma Questionnaire (WSSQ) [20] and the Weight Bias Internalization Scale (WBIS) [21]. As negative effects of WBI have been observed when controlling for BMI, a call for measures that allow for assessment of weight stigmatization across different body weight categories was made [22]. The Modified Weight Bias Internalization Scale (WBIS-M; Pearl and Puhl [23]) was consequently developed to be applied across body weight statuses to assess the full impact of this problem.

Several adaptations of the WBIS-M to other languages have been developed in the recent years. A Turkish validation was conducted with college students [24]. Two Chinese versions for children and adolescents have been developed based on the samples from Hong Kong [25] and mainland China [26]. Two different German versions are also available. The Weight Bias Internalization Scale for Children (WBIS-C), was developed for children aged 9–13 years [27] and was based on the previous German version of the WBIS [28]. A recent three-item short version (WBSI-3) has also been developed using a German sample [29]. A Spanish version of the WBIS-M has been developed with a sample of adults from the general population [30]. However, there are currently no instruments for assessing WBI in Spanish children and adolescents across different weight statuses. This study was designed to cover this deficit. The specific aims were: (1) to study the internal structure of our WBIS-M version using exploratory and confirmatory factorial analysis; (2) to study its internal consistency; (3) to study the association between the WBIS-M score and the same set of external variables analysed in the original version [23] to provide information about its validity; and (4) to study their relationships with gender and weight status.

## Methods

### Participants

**Fig. 1:**
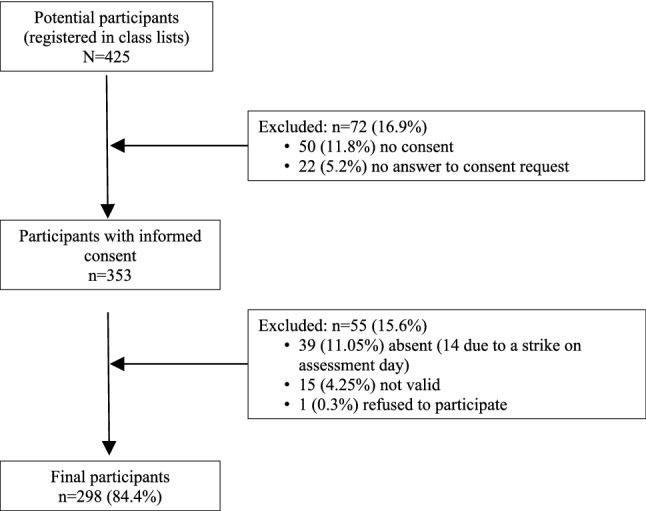
Participants’ flow diagram

This study is part of a funded project on weight bias in adolescents carried out using a representative sample of the city of Terrassa (Barcelona). Schools and classrooms were selected using a multistage cluster sampling. The final sample of this study consisted of 298 secondary school students (18.75% of the expected sample), up to the point when data collection was stopped due to the COVID-19 lockdown. No specific exclusion criteria were used and all students present at the time of the assessment with parental consent participated. Only participants who did not have parental consent, refused to participate, did not respond to the parental consent request or were invalid (because of language issues or because did not pass the surveys controls) were excluded from the original class lists. Figure 1 shows the flow diagram of the sample.

The sample came from one public and four grant-aided schools and was composed of students from the four years of Compulsory Secondary Education in the Spanish system. Table 1 shows the main characteristics of the sample.

**Table 1:** Descriptions and tests of significance for age, BMI, parental origin, weight status, DFT, Binge Eating, and WBIS-M based on comparison between genders

	Total (*n* = 298)	Female (*n* = 144)	Male (*n* = 154)	*t* (*p*)* χ^2^ (*p*)**
Age M (SD)	14.31 (1.23)	14.32 (1.28)	14.31 (1.18)	− 0.08 (.939)
BMI M (SD)	20.99 (3.49)	21.04 (3.64)	20.94 (3.35)	− 0.26 (0.796)
Parental Origin *n* (%)				
Spanish	230 (77.2)	110 (76.4)	120 (77.9)	3.80 (0.578)
European	3 (1)	2 (1.4)	1 (0.6)	
Latino	16 (5.4)	10 (6.9)	6 (3.9)	
North African	17 (5.7)	7 (4.9)	10 (6.5)	
Mixed origin	5 (1.7)	1 (0.7)	4 (2.6)	
Others	27 (9.1)	14 (9.7)	13 (8.4)	
Weight status *n* (%)				
Underweight	37 (12.4)	22 (15.3)	15 (9.7)	3.14 (0.370)
Normal weight	186 (62.4)	90 (62.5)	96 (62.3)	
Overweight	56 (18.8)	25 (17.4)	31 (20.1)	
Obesity	19 (6.4)	7 (4.9)	12 (7.8)	
EDI-3 M (SD)				
DFT (0–28)	7.67 (6.83)	9.35 (8.12)	6.10 (4.87)	− 4.20 (< 0.001)
BINGE EATING *n *(%)				
(1)	6 (2)	4 (2.8)	2 (1.3)	1.05 (0.789)
(2)	41 (13.8)	21 (14.6)	20 (13.0)	
(3)	56 (18.8)	27 (18.8)	29 (18.8)	
(4)	195 (65.4)	92 (63.9)	103 (66.9)	
WBIS-M M (SD)	2.22 (1.37)	2.57 (1.55)	1.90 (1.09)	− 4.29 (< 0.001)

(1) Binge eating + loss of control + at least once a week in the last three months + some or a lot of distress; (2) Binge eating + loss of control + at least once a month or a few times a month in the last three months, or a higher frequency but with no or little distress; (3) Binge eating + no loss of control + no distress; (4) No binge eating

*BMI* Body Mass Index, *M* Mean, *SD* Standard Deviation, *EDI-3* Eating Disorders Inventory-3, *DFT* Drive for Thinness subscale, *BINGE EATING* Binge eating severity using DSM-V criteria, *WBIS-M* Modified Weight Bias Internalization Scale

^*^Degrees of freedom for *t* test = 296

^**^Degrees of freedom for *χ*^2^: Parental Origin = 5; Weight status and Binge eating = 3

### Measures

#### Demographics

The Four-Factor Index of Social Status [31] was used to determine the socioeconomic status (SES) of the household using a weighted average of each parent’s educational and occupational level. Total scores were categorized into five different levels, namely high, medium–high, medium, medium–low and low. Height in cm and weight in kg were measured using a SECA portable stadiometer, model 214 (20–207 cm; accuracy range of 0.1 cm), and SECA portable scales, model 8777021094 (0–200 kg; accuracy range of 0.1 kg), respectively. Weight status was calculated using the World Health Organization growth reference criteria [32]. Participants also reported information about age, gender, and parental origin.

#### Weight Bias Internalization

The WBIS-M [23] was based on the original 11 items from the WBIS [21]. Items that included the word “overweight” were replaced with phrases that instead used the words “my weight”. Responses are rated on a 7-point Likert scale ranging from “Strongly Disagree” to “Strongly Agree”. Two items (1 and 9) are reverse-scored. The mean of the item responses serves as the participant’s score (range 1–7), with higher scores indicating higher internalized weight bias. The Cronbach’s alpha of the original WBIS-M was 0.94, showed strong construct validity, and presented a one-factor structure. The translation process of the WBIS-M is described in the procedures section.

We included the same set of measures as were used in the original versions of both WBIS and WBIS-M, but in their Spanish versions, to test their construct and convergent validity.

#### Anti-fat bias

The *Dislike subscale of the Anti-fat Attitudes Questionnaire* (AAQ-D) [33] in its Spanish version [34] was also administered. It has 7 items with responses ranging from 1 (‘strongly disagree’) to 7 (‘strongly agree’). Cronbach’s alpha was among 0.70 and 0.86. In our sample, Cronbach’s alpha was 0.74.

#### Self-esteem and mood

The Spanish versions of the Rosenberg Self-Esteem Scale (RSE)[35] and the Depression Anxiety Stress Scales (DASS-21)[36] were included. The Spanish version of the RSE consists of 10 items based on a 4-point scale from 1 (‘totally disagree’) to 4 (‘totally agree’). Cronbach’s alpha (0.85 to 0.88) and test–retest correlation (0.84) was found to be satisfactory. Cronbach’s alpha in our study was 0.88. In the Spanish version of the DASS-21, respondents evaluate the severity/frequency with which they have experienced each of the 21 negative emotional symptoms of depression, anxiety, and stress during the previous week on a scale from 0 to 3. Both a three-factor model and a one-factor model were used. The discriminant validity was satisfactory and Cronbach’s alfa values were 0.84, 0.70, and 0.82 for the Depression, Anxiety, and Stress subscales, respectively. Cronbach’s alpha for the whole scale in our sample was 0.92.

#### Body image and eating disorder pathology

A short 10-item Spanish version of the Body Shape Questionnaire (BSQ) [37] and the Spanish version of the Drive for Thinness (DFT) subscale of the Eating Disorders Inventory-3 [38] were included. Scores in the BSQ range from 1 (‘never’) to 6 (‘always’). This version demonstrated metric invariance and was found to be more consistent than other short versions (Warren et al. [37]). In our sample, Cronbach’s alpha was 0.91. The DFT consists of 7 items that measure restrictive tendencies in eating and weight behaviours and cognitions. The internal consistency with Cronbach’s alpha in nonclinical samples was 0.44–0.95 and test–retest values were 0.85 to 0.99. In our sample, Cronbach’s alpha was 0.82.

The original development of the WBIS-M included two questions from the Eating Disorder Diagnostic Scale (EDDS) [39] for assessing frequency of binge eating in the past three and 6 months. In our study, binge eating was measured using four previously established and validated questions used in studies focused on weight stigma in adolescents [18]. These questions were updated to align with Binge Eating Disorder criteria from DSM-V [40], assessing the presence of binge eating in the past three months (yes/no), with or without loss of control (yes/no), frequency of binge eating with loss of control (4-point scale from “every day” to “less than once per month”), and distress over binge eating (4-point scale from “not at all” to “a lot”). Based on the previous research [41], these items were combined to determine a severity score on a 4-point ordinal scale.

### Procedure

This study was supported by Terrassa City Council’s Health and Community Service. The Principal Investigator of the funded research project held meetings with the management teams of the schools to introduce the project, agree the assessment scheduling, and obtain the lists of the enrolled students. Parental consent was requested. The survey was designed in an online format on a specific platform of the company Digital Insights, and included controls as response ranges and three interspersed control questions to test attention levels. Participants who did not pass the surveys controls were excluded and their data were not registered. The missing data were avoided with this system. The survey was administered in classrooms during normal class hours, under the supervision of a group of previously trained psychology master’s students and their regular teacher. Assent from each participant was also requested. Groups of 5–7 participants were taken to a private room to take height and weight measures, following a standardized protocol [42]. The participants returned to the classroom to continue with the survey. Confidentiality was guaranteed throughout the entire process, and data processing was based on anonymous data. The assessments were conducted between March 4–12, 2020. On March 13, the COVID-19 lockdown was decreed and assessments were stopped. This study was conducted in accordance with the Declaration of Helsinki and was approved by the Ethics Committee on Animal and Human Experimentation at the *Universitat Autònoma de Barcelona* (CEAAH 3451).

The adaptation of the WBIS-M from English to Spanish was conducted following the International Test Commission Guidelines [43]. Two translators whose native tongue was the target language (Spanish) translated the original questionnaire from English to Spanish. A panel of experts on the subject, whose native language was Spanish and who had a good level in the source language (English), unified a preliminary Spanish version. Then, the Spanish version was back-translated into English by two other independent translators. A bilingual expert–reconciliation panel, made up of the translators and members of the research team, then concluded a final Spanish version that guaranteed conceptual equivalence. Last, a pilot study was carried out with fifteen participants within the age and group of interest to confirm that the final Spanish version could be read and understood. The final document did not need to be modified.

### Data analysis

The following analytic plan was pre-specified. The psychometric properties of the WBIS-M for adolescents were analysed through IBM SPSS Statistics, version 24 (IBM Corp., Armonk, N.Y., USA), and IBM SPSS Amos Statistics, version 20 (IBM Corp., Chicago, I.L., USA). A cross-validation was conducted [44], for which the sample was split into two random subsamples. Exploratory factor analysis (EFA) was conducted with subsample 1 (*n* = 149) and a confirmatory factor analysis (CFA) was conducted with subsample 2 (*n* = 149). Non-statistically significant differences were observed between samples in terms of school (*χ*^2^ = 9.061, df = 5, *p* = 0.107), year (*χ*^2^ = 2.403, df = 3, *p* = 0.493) and gender (*χ*^2^ = 0,215, df = 1, *p* = 0.643). The adequacy of the data to conduct the EFA was tested using the Kaiser–Meyer–Olkin (KMO) test and Bartlett’s sphericity test [45]. In line with the original development of the WBIS [21], the EFA was conducted through varimax rotation. In addition, the Kaiser–Guttman [46] rule and parallel analysis (Hayton, Allen & Scarpello, 2004) were applied to retain factors.

CFA was conducted through the unweighted least squares (ULS) method, because data did not fit multivariate normality. The following goodness-of-fit indices were obtained: goodness-of-fit index (GFI), the parsimony GFI (PGFI), and the normed fit index (NFI), and the standardized root-mean square residual (SRMR). The cutoff points for these indices were: equal to or higher than 0.9 for GFI, and NFI, 0.6 for PGFI, and lower than 0.08 for SRMR [47, 48]. The internal consistency of the scale was analysed using Cronbach’s alpha, McDonald’s omega, and item-test corrected correlations. According to Tavakol and Dennick [49], cutoff points for acceptable internal consistency indices range from 0.7 to 0.95. The cutoff value of 0.4 was considered for acceptable item-test correlations [50].

A descriptive analysis of the questionnaires was conducted. Spearman’s correlation (for the ordinal variable Binge Eating) and partial correlations controlled by BMI were obtained to assess the relationship between the WBIS-M and the other subscales. Moreover, the WBIS-M score was correlated to BMI. Mean comparisons (ANOVA), adjusted by parental origin, age, and socioeconomic status, were also calculated to analyse the relationship between weight status and gender and the scores obtained in the WBIS-M. It must be noted that standardised values (*z*) of BMI were considered. Simple comparisons were also obtained.

## Results

### Descriptive analysis of items

Descriptive analyses of items are provided in Table 2. It must be noted that item 8 obtained higher scores in terms of skewness and kurtosis as compared to the rest of items.

**Table 2:** Descriptive statistics of items

Item	Mean (SD)	Skewness	Kurtosis
1.Because of my weight, I feel that I am just as competent as anyone/*Dado mi peso, siento que soy tan competente como cualquier otra persona*^a^	4.71 (2.06)	− 0.44	− 1.13
2.I am less attractive than most other people because of my weight/*Soy menos atractivo/a que la mayoría de las personas debido a mi peso*	2.53 (1.79)	1.02	− 0.10
3.I feel anxious about my weight because of what people might think of me/*Me siento nervioso/a respecto a mi peso debido a lo que la gente puede pensar de mí*	2.79 (2.04)	0.78	− 0.87
4.I wish I could drastically change my weight/*Desearía poder cambiar mi peso drásticamente*	2.74 (2.07)	0.93	− 0.52
5.Whenever I think a lot about my weight, I feel depressed/*Siempre que pienso mucho sobre mi peso, me deprimo*	2.30 (1.87)	1.30	0.33
6.I hate myself for my weight/*Me odio a mí mismo/a por mi peso*	1.74 (1.51)	2.32	4.68
7.My weight is a major way that I judge my value as a person/*Mi peso es una de las principales maneras por las que juzgo mi valía personal*	1.85 (1.54)	2.09	3.71
8.I don’t feel that I deserve to have a really fulfilling social life, because of my weight/*Debido a mi peso, no siento que merezca tener una vida social que me haga feliz*	1.38 (1.13)	3.59	13.25
9.I am OK being the weight that I am/*Estoy bien con el peso que tengo*^a^	4.95 (2.09)	− 0.60	− 1.09
10.Because of my weight, I don’t feel like my true self/*Debido a mi peso, no siento que sea realmente yo mismo/a*	1.72 (1.49)	2.32	4.67
11.Because of my weight, I don’t understand how anyone attractive would want to date me/*Debido a mi peso, no entiendo cómo alguien atractivo querría tener una cita conmigo*	2.09 (1.81)	1.60	1.32

^a^Item reverse scored

Spanish translation in italics

### Internal structure

The data obtained from subsample 1 (*n* = 149) proved to be adequate for analysis through an EFA (KMO = 0.915, *χ*^2^ = 1075.633, df = 55, *p* < 0.0001). According to parallel analysis and Kaiser–Guttman rule, one-factor solution was retained, explaining the 54.57% of variance. Item 1 showed a low communality (0.092) and the lowest factor loading (0.302), whereas the rest of the items showed higher communalities and adequate factor loadings of above 0.6 in all cases (Table 3). These results suggested that item 1 did not fit adequately into the one-factor model. Moreover, item–total corrected correlation was low (0.299), whereas the rest of the items showed values higher than 0.6. Therefore, item 1 was excluded from further analysis.

**Table 3:** Communalities and factor loadings obtained from subsample 1 (*n* = 149) in the EFA

Item	Communalities	Factor loading
1.Because of my weight, I feel that I am just as competent as anyone/*Dado mi peso, siento que soy tan competente como cualquier otra persona*^a,b^	0.092	0.343
2.I am less attractive than most other people because of my weight/*Soy menos atractivo/a que la mayoría de las personas debido a mi peso*	0.565	0.752
3.I feel anxious about my weight because of what people might think of me/*Me siento nervioso/a respecto a mi peso debido a lo que la gente puede pensar de mí*	0.683	0.827
4.I wish I could drastically change my weight/*Desearía poder cambiar mi peso drásticamente*	0.645	0.803
5.Whenever I think a lot about my weight, I feel depressed/*Siempre que pienso mucho sobre mi peso, me deprimo*	0.762	0.873
6.I hate myself for my weight/*Me odio a mí mismo/a por mi peso*	0.667	0.817
7.My weight is a major way that I judge my value as a person/*Mi peso es una de las principales maneras por las que juzgo mi valía personal*	0.656	0.810
8.I don’t feel that I deserve to have a really fulfilling social life, because of my weight/*Debido a mi peso, no siento que merezca tener una vida social que me haga feliz*	0.478	0.691
9.I am OK being the weight that I am/*Estoy bien con el peso que tengo*.^a^	0.427	0.653
10.Because of my weight, I don’t feel like my true self/*Debido a mi peso, no siento que sea realmente yo mismo/a*	0.458	0.677
11.Because of my weight, I don’t understand how anyone attractive would want to date me/*Debido a mi peso, no entiendo cómo alguien atractivo querría tener una cita conmigo*	0.570	0.755
Eigenvalue	6.404	

^a^Item reverse scored

^b^Analysis did not support the inclusion of item 1

Spanish translation in italics

CFA was conducted with subsample 2 (*n* = 149) to test whether the unidimensional structure of the questionnaire could be confirmed in another set of data. The ten items of the questionnaire (excluding item 1) were considered in the analysis given that the data showed an adequate fit to the one-factor model (*χ*^2^ = 200.515, df = 35), GFI = 0.995, PGFI = 0.992, NFI = 0.991, and SRMR = 0.060). Standardised regression weights were adequate in all cases according to Floyd and Widaman [44], ranging from 0.449 to 0.880.

### Internal consistency

Reliability analyses were performed on both subsamples and the total sample. As shown in Table 4, internal consistency was higher than 0.9 in both subsamples and in the total sample, and according to Cronbach’s alpha and McDonald’s omega. Moreover, item analyses revealed that all the items contributed to the internal consistency of the scale, since item–total correlations were higher than 0.6 in both the subsamples and in the total sample. The scale showed an adequate internal consistency in terms of both the reliability coefficient and item–total correlations.

**Table 4:** Internal consistency coefficients and item–total correlations in both subsamples and the total sample

	Subsample 1 (*n* = 149)			Subsample 2 (*n* = 149)			Total sample (*n* = 298)		
	*α* (95%CI)	*ω* (95% CI)	I–T correlation range	*α* (95%CI)	*ω* (95% CI)	I–T correlation range	*α* (95%CI)	*ω* (95% CI)	I–T correlation range
WBIS-M 10-items version	0.930 (0.913–0.944)	0.936 (0.920–0.951)	0.633–0.849	0.928	0.934 (0.918 – 0.950)	0.681–0.841	0.929	0.935 (0.926 – 0.946)	0.659–0.845

*I–T* Item–total

### Correlations and differences by gender and weight status

Table 5 shows the means and standard deviations of all the scales. The mean of the WBIS-M was 2.22 (SD = 1.37). The same table shows the results of the WBIS-M correlations and the correlations reported by Pearl and Phul [23] for the original WBIS-M. The original WBIS correlations found by Durso and Latner [21] are also shown.

**Table 5:** Means, correlations, and partial correlations of WBIS and WBIS-M with study measures

Measure	Mean (SD)	WBIS Correlation^a^	WBIS-M Correlation^b^	WBIS-M Correlation	WBIS Partial Correlation^a,c^	WBIS-M Partial Correlation^b,c^	WBIS-M Partial Correlation^c^
AAQ-Dislike	1.83 (0.84)	0.31**	0.17*	0.13*	0.32**	0.22**	0.12*
BSQ	1.60 (0.84)	0.74**	0.77**	0.73**	0.75**	0.72**	0.70**
DFT	7.70 (6.80)	0.47**	0.56**	0.64**	0.48**	0.50**	0.59**
RSE	2.98 (0.65)	− 0.68**	− 0.56**	− 0.53**	− 0.67**	− 0.50**	− 0.53**
DASS21	1.58 (1.18)	0.51**	0.44**	0.40**	0.50**	0.43**	0.40**
EDDS^e^							
3Mos		0.25**	0.36**	–	0.24**	0.29**	–
6Mos		0.32**	0.47**	–	0.31**	0.40**	–
Binge Eating	–			− 0.16*,^d^	–	–	–
BMI	20.99 (3.49)	0.15	0.47**	0.31**	–	–	–

*WBIS* Weight Bias Internalization Scale, *WBIS-M* Modified Weight Bias Internalization Scale, *AAQ-Dislike* Dislike Subscale of Anti-fat Attitudes Questionnaire, *BSQ* Body Shape Questionnaire, *DFT* Drive for thinness subscale of Eating Disorders Inventory-3, *RSE* Rosenberg Self-Esteem Scale, *DASS-21* 21-item Depression Anxiety Stress Scales, *EDDS 3Mos and 6Mos* Binge eating behaviour in the past 3 and 6 months as measured by the Eating Disorder Diagnostic Scale, *Binge Eating* Binge eating severity using DSM-V criteria

^a^Statistics also reported in Durso and Latner [21]

^b^Statistics also reported in Pearl and Puhl [23]

^c^Controlling for BMI

^d^Spearman because Binge Eating is an ordinal variable

^e^Reported in Durso and Latner [21] and Pearl and Puhl [23]

^*^p < 0.05, two-tailed

^**^p < 0.01, two-tailed

A significant positive correlation was found between WBIS-M and BMI. The total and partial correlations (controlling for BMI) for all the other measures were comparable to those reported in the original validation studies, except for those corresponding to the Dislike scale, which were slightly lower [21, 23]. WBIS-M showed significant correlations with all the outcome variables when controlling for BMI.

**Fig. 2:**
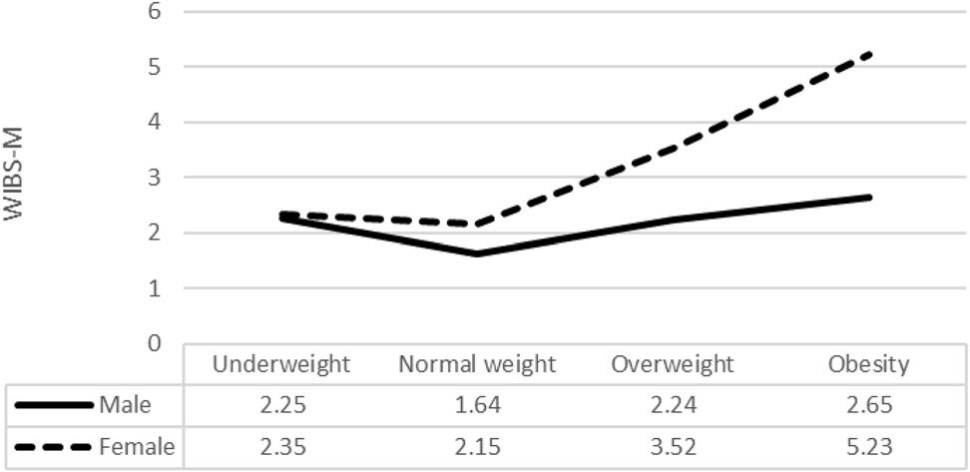
Line graph with the means of WBIS-M by gender and weight status

Figure 2 shows the WBIS-M means based on the weight status (categorized according to zBMI) and gender.

In the ANOVA, adjusted by parental origin, age, and socioeconomic status (degrees of freedom are corrected for non-homogeneity of variances), the interaction between the two variables was significant (*F*_(1, 287)_ = 6.14; *p* = 0.014; partial *η*^2^ = 0.060), as were the effects of weight status (*F*_(1, 287)_ = 22.65; *p* < 0.001; partial *η*^2^ = 0.193) and gender. WBIS-M scores were significantly higher in the girls than in the boys (*F*_(1, 287)_ = 33.47; *p* < 0.001; partial *η*^2^ = 0.104), with means of 2.57, SD = 1.55, and 1.90, SD = 1.09, respectively (Cohen’s *d* = 0,5).

A simple contrast shows that participants with obesity status (reference category) scored significantly higher on the WBIS-M than participants with underweight status (*p* < 0.001), normal weight status (*p* < 0.001), and overweight status (*p* < 0.001).

## Discussion

To the best of our knowledge, this is one of the few studies that provides a validated version of the WBIS-M for use with adolescents across weight categories, and the first one adapted to Spanish. After conducting both an EFA and a CFA of the WBIS-M with different subsamples, the results confirmed adequate psychometric properties of our WBIS-M version, with adequate fit of the 10-item version (excluding item 1), high internal consistence, and support for its validity.

The study had four specific objectives. The first was to study the internal structure of this Spanish version with adolescents using EFA and CFA. Item 1 (‘‘Because of my weight, I feel that I am just as competent as anyone”), a reverse-keyed item, obtained a low communality when compared with the rest of the items, as well as a low factor loading and a low item–total correlation. Therefore, it was not included in subsequent analyses. These results are consistent with previous studies conducted with adults who were overweight or had obesity, also suggesting that this item did not correctly fit the WBIS-M structure [18, 28, 51, 52]. A similar result was found with WBIS versions for adolescents with obesity seeking bariatric surgery [53]. Regarding WBIS-M versions, the German WBIS-C for children also removed item 1 [27], as did the Mainland Chinese version for children and adolescents [26]. In the Chinese version with children and adolescents from Hong Kong, item 1 was retained, although it showed a very low factor loading [25]. A similar result was found in the Spanish version of the WBIS-M for adults, where item 1 was retained in the final version, despite it showing the lowest factor loading and the lowest standardized regression weight [30]. The removal of item 1 from the original WBIS-M 11-item version has been observed in recent multinational studies on WBI correlates [54]. In summary, our exclusion of item 1 based on its anomalous psychometric functioning is supported by evidence coming from the previous studies. After removing this item WBIS-M yielded a one-factorial structure with adequate good fit, replicating the unidimensional structure identified in the original development of the WBIS [21] and the WBIS-M [23]. This one-factorial structure was also found in all adaptations of the WBIS-M to other languages [24–27, 29, 30].

The mean WBIS-M score was slightly below the middle range. When compared with other versions of the WBIS-M, which used the original 1–7 range scores, our mean WBIS-M score was slighly lower than in versions focused on adults, including the original version [23, 30], and similar to other versions with youth and adolescents [24, 26]. Because WBI research has mainly focused on adults, and few studies have been developed with adolescents across different weight statuses, more studies are needed to confirm this trend.

The second objective was to study the internal consistency. We obtained excellent reliability results in terms of both the internal consistency coefficient and item–total correlations in both subsamples and for the whole sample. The internal consistency was very similar to the original version [23] and similar or slightly higher than in the other WBIS-M versions published to date [24–27, 29, 30].

The third goal was to study the association between the WBIS-M score and the same set of external variables analysed in the original version. We found that higher scores of WBIS-M were positively associated with an increase in dislike of people with obesity, higher body dissatisfaction and drive for thinness, lower self-esteem, higher symptoms of stress, depression and anxiety, and a higher frequency and severity of binge eating behaviour. Total and partial correlations (adjusted by BMI) were highly comparable to those reported in the original version, except the total correlation for the binge eating measure, which was lower in our study. Nevertheless, the fact that we used the Spanish versions of the same measures used in the original version, except in the case of binge eating, for which we used the stricter DSM-V criteria [40], and that this variable was analysed as ordinal (and not as quantitative as in the original), must be taken onto account. The original version of the WBIS-M [23] used the same measures as per the original version of the WBIS [21], and DSM-V criteria were not available when the WBIS was originally developed. All the correlated variables have been considered as correlates of WBI in adolescents [18, 53, 55–57]. These results provide support for the validity of the questionnaire.

The fourth and last goal of this study was to assess the relationships between WBI, gender, and weight status. Higher scores of WBI were found in girls and in higher weight statuses. Social pressure to attain the beauty ideal is higher among young women than among young men [58], which could make women more vulnerable to weight-based stigmatization [59]. Furthermore, young people who are overweight or are living with obesity are more prone to experience weight-based discrimination [6]. Our results are in line with research in adults across weight categories showing that women and people with higher BMIs show higher WBI levels than men and people with lower BMIs [23, 54]. Regarding children and adolescents, a higher risk of reporting weight discrimination has been associated with the female gender and those with a higher BMI in Chinese adolescents [57]. Higher WBI scores in girls and participants who were overweight than in boys and participants who were not overweight have been reported in previous research conducted in Germany with a general population sample [27]. The same results were observed in another German study conducted with adolescents seeking weight loss [56]. In contrast, other studies conducted with adolescents from the USA did not find gender differences regarding WBI in adolescents [18, 53]. Notably, these last studies were conducted with samples of adolescents seeking weight loss or bariatric surgery. Differences in sample characteristics could explain these results. More studies are needed to clarify the role of gender regarding WBI in adolescents. Together, our results regarding WBI and gender and weight status differences support the known-groups validity of our WBIS-M version.

### Strength and limits

This study has several limitations and strengths. Regarding limitations, there is a lack of data on the test–retest reliability. Although studies conducted with adults have provided stability data for the WBIS-M (e.g. Macho et al. [30]), up to now only the Mainland Chinese version with a general population sample [26] and the German version with participants seeking weight loss [56] have reported satisfactory stability with adolescent samples. Another limitation is related to the sample. A bigger sample would provide a more accurate analysis of the internal structure of the questionnaire. Moreover, it has to be noted that the sample is not representative of Spanish adolescents. Last, measurement invariance by relevant groups such as age, gender, or weight status is necessary and this could not be estimated in our study because of the insufficient sample size. Measurement invariance would provide substantial endorsement for using the same version of the questionnaire with different groups (e.g., by gender, different ages, weight status) without targeted adaptations to specific group characteristics, and for detecting real differences in WBI across these groups that are not attributable to different interpretations of WBIS-M item content between groups. To date, only the German WBIS-C [27] and the WBIS-M version with Chinese adolescents from Hong Kong [25] have provided invariance for gender groups, and the latter only partially for weight status. We are planning to study measurement invariance for gender, age groups, and weight status once the total sample initially conceived is assessed next year, if the restrictions derived from the COVID-19 epidemic have been lifted.

On the other hand, the study has several strengths. This is one of the few studies to provide a validated version of the WBIS-M for use with adolescents across weight categories, and the first one adapted to Spanish. The internal structure of our WBIS-M version was analysed by means of both EFA and CFA in different subsamples. The translation process was done following the International Test Commission Guidelines [43]. To ensure the quality of data collection, the survey was designed in an online format, included response ranges and three control interspersed questions, and was administered in classrooms under the supervision of a group of technicians previously trained. And last, the adolescents’ weight and height were measured objectively using portable and accurate instruments, and following a standardized protocol, preventing inaccuracy in the self-reported data in adolescents [60, 61].

## Conclusion

Our findings provide support for the validity and reliability of our WBIS-M version for use with adolescents across weight categories. The availability of a Spanish version of the WBIS-M for adolescents will provide research teams and practitioners from Spain who are addressing weight bias in the population with a validated version of one of the few existing but the most employed instrument for assessing WBI. In addition to its value for research and clinical settings, data coming from the use of this new tool could contribute as evidence to support the promotion of educational, regulatory, and legal initiatives designed to prevent weight stigma and discrimination, as recommended by a recent international consensus statement for ending obesity stigma [2].

### What is already known on this subject?

Weight bias internalization (WBI) is pervasive and potentially damaging for health, independent of weight status. The WBIS-M is one of the few existing but the most employed instrument for assessing WBI, and several validations to different languages have been developed. There are currently no Spanish versions for assessing WBI in children and adolescents across different weight statuses.

### What this study adds?

This study provides research teams and practitioners from Spain who are addressing weight bias in the population with a validated version of one of the few existing but the most employed instrument for assessing WBI among adolescents across different weight statuses.

## Data Availability

The data presented in this study are available on request from the corresponding author. The data are not publicly available due to privacy or ethical reason.
